# Molecular investigation of an outbreak associated with total parenteral nutrition contaminated with NDM-producing *Leclercia adecarboxylata*

**DOI:** 10.1186/s12879-021-05923-0

**Published:** 2021-02-28

**Authors:** Elvira Garza-González, Paola Bocanegra-Ibarias, Eduardo Rodríguez-Noriega, Esteban González-Díaz, Jesús Silva-Sanchez, Ulises Garza-Ramos, Iván Fernando Contreras-Coronado-Tovar, José Ecil Santos-Hernández, David Gutiérrez-Bañuelos, Juan Pablo Mena-Ramirez, Saúl Ramírez-De-los-Santos, Adrián Camacho-Ortiz, Rayo Morfín-Otero

**Affiliations:** 1 Hospital Universitario Dr. José Eleuterio González, Universidad Autónoma de Nuevo León, Monterrey, Nuevo León Mexico; 2 Hospital Civil de Guadalajara Fray Antonio Alcalde, Instituto de Patología Infecciosa y Experimental, Centro Universitario de Ciencias de la Salud, Universidad de Guadalajara, Guadalajara, Jalisco Mexico; 3 Centro de Investigación Sobre Enfermedades Infecciosas, Instituto Nacional de Salud Pública, Cuernavaca, Morelos Mexico; 4 Hospital de Pediatría de Centro Médico Nacional de Occidente, Guadalajara, Jalisco Mexico; 5 Hospital General de Zona No.21 IMSS, Centro Universitario de los Altos (CUALTOS), Universidad de Guadalajara, Tepatitlán de Morelos, Jalisco Mexico; 6 Instituto de Investigación en Biociencias, Centro Universitario de los Altos, Universidad de Guadalajara, Tepatitlán de Morelos, Jalisco Mexico

**Keywords:** NDM-carrying Leclercia adecarboxylata, Outbreak of *L. adecarboxylata*, Carbapenem-resistant *L. adecarboxylata*, Contaminated total parenteral nutrition, Bloodstream infections

## Abstract

**Background:**

This study aimed to determine the epidemiological, microbiological, and molecular characteristics of an outbreak of carbapenem-resistant *Leclercia adecarboxylata* in three hospitals associated with the unintended use of contaminated total parental nutrition (TPN).

**Methods:**

For 10 days, 25 patients who received intravenous TPN from the same batch of a formula developed sepsis and had blood cultures positive for *L. adecarboxylata*. Antimicrobial susceptibility and carbapenemase production were performed in 31 isolates, including one from an unopened bottle of TPN. Carbapenemase-encoding genes, extended-spectrum β-lactamase–encoding genes were screened by PCR, and plasmid profiles were determined. Horizontal transfer of carbapenem resistance was performed by solid mating. Clonal diversity was performed by pulsed-field gel electrophoresis. The resistome was explored by whole-genome sequencing on two selected strains, and comparative genomics was performed using Roary.

**Results:**

All 31 isolates were resistant to aztreonam, cephalosporins, carbapenems, trimethoprim/sulfamethoxazole, and susceptible to gentamicin, tetracycline, and colistin. Lower susceptibility to levofloxacin (51.6%) and ciprofloxacin (22.6%) was observed. All the isolates were carbapenemase producers and positive for *bla*_NDM-1_, *bla*_TEM-1B_, and *bla*_SHV-12_ genes. One main lineage was detected (clone A, 83.9%; A1, 12.9%; A2, 3.2%). The *bla*_NDM-1_ gene is embedded in a Tn*125*-like element.

Genome analysis showed genes encoding resistance for aminoglycosides*,* quinolones*,* trimethoprim, colistin*,* phenicols, and sulphonamides and the presence of IncFII (Yp), IncHI2, and IncHI2A incompatibility groups. Comparative genomics showed a major phylogenetic relationship among *L. adecarboxylata* I1 and USDA-ARS-USMARC-60222 genomes, followed by our two selected strains.

**Conclusion:**

We present epidemiological, microbiological, and molecular evidence of an outbreak of carbapenem-resistant *L. adecarboxylata* in three hospitals in western Mexico associated with the use of contaminated TPN.

**Supplementary Information:**

The online version contains supplementary material available at 10.1186/s12879-021-05923-0.

## Background

Total parenteral nutrition (TPN) is a nutritional supplement for patients unable to receive oral or enteral nutrition. TPN indications mainly include the presence of chronic intestinal obstruction, bowel pseudo-obstruction with food intolerance in infants with an immature gastrointestinal system or a congenital gastrointestinal malformation, among others [1] Because manipulation of these supplements may enhance the risk for microbial contamination, absolute compliance to good manufacturing practices is required [2–4]. When these practices are not strictly followed, TPN-related outbreaks may occur, commonly leading to sepsis with high mortality [5].

*Leclercia adecarboxylata* is a gram-negative rod with a phenotypic resemblance to *Escherichia coli,* and Lecler first described it in 1962 as *Escherichia adecarboxylata.* In 1986 based on DNA hybridization studies, this species was reassigned as part of the *Enterobacterales* order, *Enterobacteriaceae* family, and *Leclercia* genus [6].. This bacterial species has global distribution in a variety of foods, water, and animals and exists as a commensal organism in the gut [7], and has been associated with bacteremia and wound infections and peritonitis, pneumonia, and other infections [8].

Although *L. adecarboxylata* is usually susceptible to antimicrobials, isolates have been reported with resistance to cephalosporins due to extended-spectrum β-lactamase (ESBL) production [9]. Additionally, *L. adecarboxylata* carbapenem-resistant isolates harboring the *bla*_NDM_ gene have been reported in China and Spain [10, 11]. Also, a VIM-1 metallo-β-lactamase has been reported in an isolate of *L. adecarboxylata* from a non-clinical sample [12].

This study aimed to determine the epidemiological, microbiological, and molecular characteristics of an outbreak of carbapenem-resistant *L. adecarboxylata* in three hospitals in western Mexico associated with the unintended use of contaminated TPN.

## Methods

### Hospital settings

This report includes the cases reported by three hospitals in the state of Jalisco: Hospital Civil de Guadalajara Fray Antonio Alcalde (HC-FAA), Hospital de Pediatría del Centro Médico Nacional de Occidente (HP-CMNO), and Hospital General de Zona 21 (HGZ-21).

The HCG is a 1000-bed tertiary care teaching hospital with a daily occupancy rate of 95%. The HPCMNO is a 205-bed tertiary care teaching hospital with a daily occupancy rate of 93%. Both hospitals serve adult and pediatric populations from the Guadalajara metropolitan area (approximately 4.0 million people) and surroundings states. The HGZ is a 73-bed secondary care hospital with a daily occupancy rate of 97%. This hospital attends most of the Tepatitlán (a city 73 Km from Guadalajara) population with social security (170,701 people). All three hospitals treat only acute conditions.

### Epidemiological investigation

An outbreak started on May 17, when the bacteriology laboratory reported eight blood cultures positive for *L. adecarboxylata*. For 10 days, 25 patients who received intravenous TPN fluids developed sepsis (fever, chills, worsening clinical condition, and leukocytosis) and had blood cultures positive for *L. adecarboxylata*.

The isolation of the same unusual species from patients at several localities indicated that an outbreak was occurring, and an investigation was launched. The finding led the Ministry of Health (Secretaria de Salud) to trigger a nationwide alert, leading to the immediate withdrawal of the TPN formula by the manufacturer.

The implicated vehicles of infection were one batch of TPN formula, supplied in 500-mL bottles. The outbreak was over by May 27, 2019, when the last case presented, and no new cases appeared after.

During the outbreak, a total of 30 *L. adecarboxylata* isolates were recovered from 25 patients. Additionally, an extensive epidemiological investigation was conducted, tracing the batch number of TPN formula used in all patients, and *L. adecarboxylata* was isolated in one sealed, unopened bottle of TPN belonging to the same batch administered to the patients. Furthermore, different equipment parts such as bags, connectors, ventilators, and others were submitted to standard microbiological sterility tests, and no microorganisms were recovered. All isolates collected were sent to a reference laboratory for further analysis.

### Identification and antimicrobial susceptibility testing

Identification of isolates was confirmed by matrix-assisted laser desorption ionization time-of-flight mass spectrometry using the Bruker Biotyper system (Bruker Daltonics, Germany) as described previously [13]. Fresh colonies from each strain were spotted onto MALDI-TOF target plates; the spots were covered with 1 μl of 70% formic acid and air-dried. Each spot was overlaid with 1 μl of a matrix solution α-cyano-4-hydroxycinnamic acid 10 mg/mL (Sigma Aldrich, Missouri*,* United States). Samples in spots were analyzed by the Bruker Microflex LT system [14]. The antimicrobial susceptibility was performed by the broth microdilution method using the Clinical & Laboratory Standards Institute (CLSI) guidelines [15]. Carbapenemase production was detected using the CarbaNP test, according to the CLSI [15].

### Molecular methods

Carbapenemase-encoding genes (*bla*_KPC-type_, *bla*_VIM-type_, *bla*_IMP-type_, *bla*_NDM-type_, *bla*_OXA-48-type_) and ESBL–encoding genes (*bla*_TEM-type_, *bla*_SHV-type_, *bla*_CTX_-_type_) were screened by PCR [16]. Horizontal transfer of carbapenem resistance was performed by solid mating using *Escherichia coli* J53–2 as the recipient strain [17, 18]. Transconjugants were selected on Luria-Bertani (LB) agar supplemented with rifampicin (100 μg/mL) plus cefoxitin (30 μg/mL). When the conjugation was unsuccessful, the assay was performed in LB agar supplemented with rifampicin (100 μg/mL) plus ceftazidime (30 μg/mL). The plasmid profile was obtained from the *L. adecarboxylata* isolates and transconjugants, according to the method described by Kieser [19].

Clonal diversity was performed using pulsed-field gel electrophoresis following the protocol and conditions previously described using the enzyme *SpeI* (Takara Bio Inc., Shiga Japón) [20] and interpretation from Tenover et al. criteria [21].

### Whole-genome sequencing

Whole-genome sequencing (WGS) was performed in two selected isolates. DNA was extracted using the DNeasy kit (Qiagen, Germany) according to manufacturer instructions. Sequencing was performed using the Illumina (NextSeq 500) platform. Quality-based trimming was performed with the Trim Galore software, and de novo assembly was completed with SPAdes v3.12.0. The draft genomes were annotated using the NCBI Prokaryotic Genome Annotation Pipeline. The genetic context of the *bla*_NDM-1_ gene was determined by in silico analysis.

### Comparative genomics

The comparative genomics *L. adecarboxylata* genomes included our 16,342 and 16,400 strains, and the deposited USDA-ARS-USMARC-60222, R25, and I1 genomes obtained from a bull, a rabbit, and a human, respectively.

Comparative genomics was performed with the suite Roary using default parameters [22]. The phylogenetic tree’s construction based on Roary’s core genes alignment was constructed with RAxML v.8 under the GTRGAMMA model [23]. The phylogeny, the pan-genome presence, absence information, and associated metadata were visualized in the Phandango viewer [24]. The ResFinder 3.2 and PlasmidFinder 2.1 tools (http://www.genomicepidemiology.org) were used to identify acquired genes, chromosomal mutations for antibiotic resistance (resistome), and replicon typing of plasmids. In addition, the class 1 integrons were identified using the WGS data on the Integron database (INTEGRAL) (http://integrall.bio.ua.pt/?).

## Results

### Affected population and outcome

All patients, the majority of whom were children (mean age, 78 months or 6.4 years), had been administered TPN via the central line. One patient died (Table 1, supplementary Table 1). The population affected include 23 children and two adults from one of the hospitals. The mean length of stay after a positive culture was 31.2 days, range 3 to 135 days. Before a positive culture, an initial antibiotic more frequently used includes carbapenems, cephalosporins, and aminoglycosides. The termination of TPN and the use of tigecycline was effective in 24/25 patients.

**Table 1:** Demographic and clinical characteristics of patients affected by the contaminated TPN outbreak in western Mexico

Characteristic	No. (% of patients)^a^			
	Total	HC-FAA	HP-CMNO	HGZ-21
No. of patients	25	11	1	13
Mean age, months (range)	78 (1–996)	152 (1–996)	4	21 (2–168)
Male	16 (64)	5 (45.5)	1 (100)	10 (76.9)
Hospital ward				
Neonatal ICU	19 (76)	8 (72.7)	1 (100)	10 (76.9)
Pediatric surgery	4 (16)	1 (9.1)	0 (0)	3 (23.1)
General surgery	1 (4)	1 (9.1)	0 (0)	0 (0)
Internal medicine	1 (4)	1 (9.1)	0 (0)	0 (0)
Previous hospitalization	8 (32)	2 (18.2)	0 (0)	6 (46.1)
Mean LOS (days)	53.8 (15–252)	49.1 (15–135)	32	59.4 (30–252)
Mean LOS to prior positive culture (days)	22.5 (1–249)	10.4 (1–22)	14	33.5 (1–249)
Mean LOS after positive culture (days)	31.2 (3–135)	39.6 (5–135)	18	25.1 (3–31)
UCI stay	20 (80)	7 (63.6)	1 (100)	12 (92.3)
Previous surgery	10 (40)	1 (9.1)	0 (0)	9 (69.2)
Number of antibiotics used before positive culture				
1–3	15 (60)	6 (54.5)	1 (100)	8 (61.5)
4 or more	10 (40)	5 (45.5)	0 (0)	5 (38.5)
Antibiotics used before positive culture, n (%)				
Carbapenems	18 (72)	5 (45.5)	0 (0)	13 (100)
Cephalosporins	15 (60)	11 (100)	1 (100)	3 (23.1)
Aminoglycoside	13 (52)	8 (72.7)	1 (100)	4 (30.8)
Glycopeptide	7 (28)	3 (27.3)	0 (0)	4 (30.8)
Fluoroquinolones	6 (24)	0 (0)	0 (0)	6 (46.1)
Penicillin	5 (20)	4 (36.4)	0 (0)	1 (7.7)
Colistin	4 (16)	1 (9.1)	0 (0)	3 (23.1)
Metronidazole	3 (12)	2 (18.2)	0 (0)	1 (7.7)
Other	7 (28)	1 (9.1)	0 (0)	6 (46.1)
Mortality	1 (4)	1 (9.1)	0 (0)	0 (0)

*Abbreviations*: *HC-FAA* Hospital Civil Fray Antonio Alcalde, *HP-CMNO* Hospital de Pediatría del Centro Médico Nacional de Occidente, *HGZ-21* Hospital General de Zona 21, *ICU* intensive care unit, *LOS* length of stay, *TPN* total parenteral nutrition

^a^Mean (range) if otherwise noted

### Microbiology

All 31 *L. adecarboxylata* isolates were resistant to aztreonam, cefepime, cefotaxime, cefoxitin, ceftazidime, ceftriaxone, ertapenem, imipenem, meropenem, and trimethoprim/sulfamethoxazole. All the strains were susceptible to gentamicin, tetracycline, colistin, lower susceptibility to levofloxacin (51.6%), and ciprofloxacin (22.6%) was observed.

### Molecular assays

All 31 isolates [clinical (*n* = 30) and TPN formula (*n* = 1)] were carbapenemase producers and were positive for *bla*_NDM-1_*, bla*_TEM-type_, and *bla*_SHV-type_ genes, and negative for the other β-lactamases genes tested. Clinical and TPN isolates harbored three plasmids with approximate sizes of 68-, 124-, and 150-kb. The mating experiments were analyzed and were successful in all isolates; the *bla*_NDM-1_ gene was transferred onto a conjugative plasmid with an approximate 124-kb size.

The genetic relationships of 31 strains isolated indicated one main lineage highly related to the outbreak with three restriction patterns detected: A, (83.9%, 26/31), A1 (12.9%, 4/31), and A2 (2.2%, 1/31), with a percentage of similarity ranging from 90 to 100%.

### Resistome identified by whole-genome sequencing

The WGS was performed in two selected isolates: 16342 (strain A) and 16,400 (strain B), both strains from patients, and generated a total of 2,893,824 (A) and 2,406,492 (B) pair-end reads with a length of 75 bp. In total, 131 (A) and 150 (B) contigs with an N50 of 93,591 bp (A) and 108,425 bp (B) were obtained. The estimated size genome sized were 5,232,567 bp (A) and 5,299,868 bp (B) with 120× coverage.

The resistome on both strains had genes encoding resistance for aminoglycosides (*aac[6′]-Ib3, aadA2b, aph[3″]-Ib, aph[3′]-Ia* and *aph[6]-Id*), β-lactam (*bla*_NDM-1_, *bla*_TEM-1B_, and *bla*_SHV-12_), quinolones (*aac[6′]-Ib-cr*)*,* trimethoprim (*dfrA19*), colistin (*mcr-9*), phenicols *(catA2)*, and sulphonamides (*sul1*) (Supplementary Table 2). Both strains A as B the genetic context revealed the ΔIS*Aba125*-NDM-1-*bleMBL*-Δ*trpF-dsbD-cutA-*Δ*groES-groEL-*Δ*isnE* structure.

Additionally, the A strain included the *qnrS2* gene (encoding resistance for nalidixic acid), and the B strain included the *qnrB2* gene (encoding resistance for nalidixic acid) and the *dfrA12* gene (encoding resistance for trimethoprim). The class 1 integron identified showed aminoglycoside (*aadA2* and, *aacA4*) and trimethoprim (*dfrA12*) resistance genes. *dfrA12* is part of a new gene arrangement of the integron In1982 and the *gcuF*Δ23, a gene cassette of unknown function (Supplementary Table 3).

The replicon typing of plasmid analysis identified in both genomes had the IncFII (Yp) (99.13% identity), IncHI2 (100% identity), and IncHI2A (100% identity) incompatibility groups (Supplementary Table 4).

### Genomic comparison of *L. adecarboxylata* genomes

**Fig. 1:**
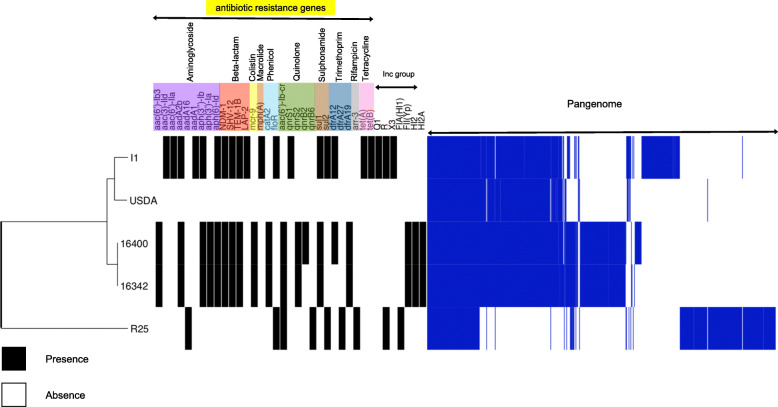
Comparative genomics of *L. adecarboxylata* genomes with their phylogenetic relationship and resistome showed as metadata. 16,342 (JACXBN000000000), 16,400 (CP060824), USDA (NZ_CP013990), I1 (NZ_MUFS00000000) and R25 (NZ_CP035382)

A major phylogenetic relationship was observed among I1 and USDA-ARS-USMARC-60222 isolates, followed by 16,342 (strain A) and 16,400 (strain B) (Fig. 1). However, the resistome identified in the genome in silico is different among these isolates. In the I1 and A, and B genomes, a large number of resistance genes were identified (Fig. 1). The incompatibility group was the same in A, B, and R25 and different in the I1 genome (carrying NDM-1) (Fig. 1).

## Discussion

Several reports have described sepsis outbreaks associated with substances administrated to patients involving *Staphylococcus saprophyticus, Enterobacter* spp., *Acinetobacte*r spp., *Pantoea agglomerans, Burkholderia cepacia,* and *Candida albicans* [25–28]. Among these outbreaks, one of the most frequently reported causes is TPN’s use, with most of them being related to the colonization of intravenous cannula. Outbreaks of infection are rarely associated with TPN products contaminated before they reached the patient. No previous report has described outbreaks associated with the use of contaminated TPN and *L. adecarboxylata*, thus demonstrating our report’s novelty. Additionally, we verified by molecular methods that the isolates collected from patients and the one from the unopened bottle were one main lineage highly related to the outbreak, with a clone A and two subtypes.

Bloodstream infection incidence in patients receiving TPN has been reported to be as high as 39% [29]*. In our study, m*ost affected patients were children, and most of them were in the neonatal intensive care unit (ICU) ward. It has been described that the administration of TPN through parenteral catheter represents a risk of 4.69 for bloodstream infections in neonates [30, 31]. In our study, unfortunately, one patient died of sepsis.

A review of 74 cases of infections associated with *L. adecarboxylata* (including bacteremia) showed high susceptibility to antibiotics, and treatment was efficient in most cases [8]. Two pediatric cases of cellulitis and urinary tract infection associated with *L. adecarboxylata* were also successfully treated with antibiotics. These reports highlight the importance of this outbreak because clinical isolates related to this outbreak were highly resistant to antibiotics and the need to become more aware of its threat in pediatric and adult populations.

Most of the patients were receiving carbapenems and cephalosporins before the outbreak, and according to microbiological results, the *L. adecarboxylata* isolates were resistant to these drugs, which may have helped to increase the severity of the outbreak.

The genetic context of the *bla*_NDM-1_ identified in this study was identical to the Tn125-like described on a pP10164-DNM plasmid from *L. adecarboxylata* P10164 isolates described previously in China [10].

Infections caused by carbapenemase-producing bacteria are difficult to treat, with the selection of initial or definitive appropriate therapy being problematic [32, 33]. The driving force for the development of resistant bacteria is the inappropriate use of antibiotics, a factor that can be forestalled with the use of sustained antimicrobial stewardship [34, 35], and the early identification and containment are crucial to help efforts [34]. A recent report looking at antimicrobial resistance in Mexico derived from 47 centers in 20 states, during 6 months in a total sample of 22,943 strains analyzed, found carbapenem resistance in 12.5% of *Klebsiella* sp. and *Enterobacter* sp., and 40% in *Pseudomonas aeruginosa* [36].

The class 1 integrons identified in *L. adecarboxylata* genomes contain genes related to aminoglycoside resistance, resulting in a new gene array in the In1982 class 1 integron. Integrons’ presence has been broadly described among *Enterobacterales* isolates, which use site-specific recombination to move resistance genes between defined sites [37, 38]. Also, mobile elements are involved in the spread of integrons [39].

Interestingly, both strains had genes encoding aminoglycoside resistance but were susceptible to gentamicin and had the *mcr-9* gene and were susceptible to colistin. Previous studies have identified *mcr*-4-bearing plasmids in *L. adecarboxylata* from human gut microbiota [10, 40], but no study had reported the association of the *mcr* genotype and the colistin resistance in this bacterial species. These findings highlight the need for epidemiological-molecular studies because strains may render as drug-resistant organisms by the selective pressure exerted by antibiotics.

Although 16,342 (strain A) and 16,400 (strain B) genomes were identified as one clone using PFGE, they showed slight differences in their pan-genome at the genomic level. Thus, the *qnrA* and *dfrA12* genes were absent in the A genome.

The A and B genomes showed an MDR resistome related to isolates from clinical settings, as was the case for the human collected genome I1.

In a recent study that included 34 *L. adecarboxylata* isolates collected from 2005 to 2017, 18 were considered clinically significant pathogens, and 16 were considered contaminants [41]; showing that this organism may be easily isolated as contaminants. In pour study, the isolates were collected from blood, so there were clinically significant.

Our study has some limitations because only two strains were sequenced, and surveillance was not performed in all hospitals that received the same batch of bottles.

## Conclusions

Outbreaks are most likely to occur when hospital staff does not adhere to basic hygiene measures. In our study, the contamination observed was detected in unopened TPN bottles, and the strain from the new bottle was identified by molecular methods to match the strain isolated from patients. Our results reinforce the importance of an in-depth epidemiological, microbiological, and molecular characterization before attributing an outbreak to a hospital’s nonadherence to hygiene measures. Continuous adherence to hygiene protocols and surveillance of unopened bottles may help to reduce outbreaks.

## Supplementary Information

**Additional file 1: S1 Table.** Demographic characteristic of patients.

**Additional file 2: S2 Table.** Antibiotics resistance family genes identified in the genomes of *L. adecarboxylata* included in the analysis.

**Additional file 3: S3 Table.** Class 1 integron identified in the *L. adecarboxylata* 16,342 and 16,400 genomes.

**Additional file 4: S4 Table.** Incompatibility groups identified in the genomes of *L. adecarboxylata* included in the analysis.

## Data Availability

The datasets generated and/or analyzed during the current study are available from GenBank under the accession numbers: JACXBN000000000, CP060824, CP013990, MUFS00000000, NZ_CP035382.

## Abbreviations

TPN: Total Parenteral Nutrition
HC-FAA: Hospital Civil Fray Antonio Alcalde
HP-CMNO: Hospital de Pediatria del Centro Medico Nacional de Occidente
HGZ-21: Hospital General de Zona 21
ICU: Intensive Care Unit
LOS: Length of stay
WGS: Whole-genome sequencing
