# Supplementary material for: Molecular investigation of an outbreak associated with total parenteral nutrition contaminated with NDM-producing Leclercia adecarboxylata
Source: BMC Infect Dis. 2021 Feb 28;21:235. doi: 10.1186/s12879-021-05923-0 (PMC7916303; doi:10.1186/s12879-021-05923-0)
Supplement: Supplementary file 1 — Additional file 1: S1 Table. Demographic characteristic of patients. [file 12879_2021_5923_MOESM1_ESM.docx]

**Supplementary table 1.-** Demographic characteristic of patients

| **Patient** | **Hospital** | **Age range (months)** | **Hospital ward** | **Primary diagnosis** | **Previous hospitalization** | **LOS (days)** | **LOS to prior positive culture** | **LOS after positive culture** | **Number of antibiotics used before positive culture** | **Clinical outcome** |
| --- | --- | --- | --- | --- | --- | --- | --- | --- | --- | --- |
| 1 | HC-FAA | 1-12 | Neonatal ICU | Sepsis | No | 26 | 7 | 19 | 4 | Cure |
| 2 | HC-FAA | 1-12 | Neonatal ICU | Sepsis | No | 30 | 0 | 33 | 3 | Cure |
| 3 | HC-FAA | 1-12 | Neonatal ICU | Sepsis | No | 20 | 0 | 20 | 2 | Cure |
| 4 | HC-FAA | 1-12 | Neonatal ICU | Sepsis | No | 53 | 19 | 34 | 3 | Cure |
| 5 | HC-FAA | 1-12 | Neonatal ICU | Sepsis | No | 18 | 0 | 18 | 2 | Cure |
| 6 | HC-FAA | 1-12 | Neonatal ICU | Sepsis | No | 135 | 3 | 135 | 4 | Cure |
| 7 | HC-FAA | >24 | General surgery | Sepsis | Yes | 79 | 17 | 61 | 4 | Cure |
| 8 | HC-FAA | >24 | Internal medicine | Sepsis | Yes | 27 | 22 | 5 | 5 | Death |
| 9 | HC-FAA | 1-12 | Pediatric surgery | Sepsis | No | 15 | 9 | 6 | 3 | Cure |
| 10 | HC-FAA | 1-12 | Neonatal ICU | Sepsis | No | 68 | 19 | 50 | 3 | Cure |
| 11 | HC-FAA | 1-12 | Neonatal ICU | Sepsis | No | 69 | 15 | 55 | 6 | Cure |
| 12 | HC-FAA | 1-12 | Neonatal ICU | Respiratory distress | No | 32 | 14 | 18 | 2 | Cure |
| 13 | HGZ-21 | 1-12 | Neonatal ICU | Hemodynamic repercussions | No | 33 | 2 | 31 | 2 | Cure |
| 14 | HP-CMNO | 1-12 | Neonatal ICU | Diaphragmatic hernia | No | 30 | 0 | 24 | 3 | Cure |
| 15 | HP-CMNO | 1-12 | Neonatal ICU | Hemodynamic repercussions | No | 39 | 8 | 31 | 4 | Cure |
| 16 | HP-CMNO | 1-12 | Neonatal ICU | Coarctation of the aorta | No | 45 | 20 | 24 | 2 | Cure |
| 17 | HP-CMNO | 1-12 | Neonatal ICU | Cholestatic syndrome | Yes | 33 | 2 | 31 | 3 | Cure |
| 18 | HP-CMNO | 1-12 | Neonatal ICU | Intestinal atresia type 1 | Yes | 41 | 13 | 23 | 4 | Cure |
| 19 | HP-CMNO | 1-12 | Neonatal ICU | Hirschsprung disease | Yes | 40 | 23 | 17 | 4 | Cure |
| 20 | HP-CMNO | 1-12 | Neonatal ICU | Covulsive crisis | No | 61 | 31 | 30 | 3 | Cure |
| 21 | HP-CMNO | 1-12 | Neonatal ICU | Pulmonary atresia | Yes | 39 | 8 | 31 | 5 | Cure |
| 22 | HP-CMNO | 1-12 | Neonatal ICU | Urinary tract infection | No | 68 | 42 | 26 | 3 | Cure |
| 23 | HP-CMNO | >24 | Pediatric surgery | Tumor | Yes | 46 | 17 | 29 | 4 | Cure |
| 24 | HP-CMNO | >24 | Pediatric surgery | Chemical poisoning | No | 45 | 19 | 26 | 3 | Cure |
| 25 | HP-CMNO | 13-24 | Pediatric surgery | Short bowel syndrome | Yes | 252 | 249 | 3 | 3 | Cure |

Abbreviations: HC-FAA, Hospital Civil Fray Antonio Alcalde; HGZ-21, Hospital General de Zona 21; HP-CMNO, Hospital de Pediatría del Centro Médico Nacional de Occidente; M: Male; F: female; ICU, intensive care unit; LOS, length of stay.
