# Supplementary material for: Molecular investigation of an outbreak associated with total parenteral nutrition contaminated with NDM-producing Leclercia adecarboxylata
Source: BMC Infect Dis. 2021 Feb 28;21:235. doi: 10.1186/s12879-021-05923-0 (PMC7916303; doi:10.1186/s12879-021-05923-0)
Supplement: Supplementary file 2 — Additional file 2: S2 Table. Antibiotics resistance family genes identified in the genomes of L. adecarboxylata included in the analysis. [file 12879_2021_5923_MOESM2_ESM.docx]

Supplementary table 2. Antibiotics resistance family genes identified in the genomes of *L. adecarboxylata* included in the analysis.

| Antibiotic family | Gene | 16342 | **16400** | **USDA-ARS-USMARC-60222** | **I1** | **R25** |
| --- | --- | --- | --- | --- | --- | --- |
| aminoglycoside | *aac(6')-Ib3* | ***+*** | ***+*** | *-* | *-* | *-* |
| aminoglycoside | *aac(3)-Ild* | *-* | *-* | *-* | ***+*** | *-* |
| aminoglycoside | *aac(6')-Ila* | *-* | *-* | *-* | ***+*** | *-* |
| aminoglycoside | *aadA2b* | ***+*** | ***+*** | *-* | *-* | *-* |
| aminoglycoside | *aadA2b* | *-* | *-* | *-* | ***+*** | *-* |
| aminoglycoside | *aadA16* | *-* | *-* | *-* | *-* | ***+*** |
| aminoglycoside | *aadA1* | *-* | *-* | *-* | ***+*** | *-* |
| aminoglycoside | *aph(3'')-Ib* | ***+*** | ***+*** | *-* | ***+*** | *-* |
| aminoglycoside | *aph(3')-Ia* | ***+*** | ***+*** | *-* | *-* | *-* |
| aminoglycoside | *aph(6)-Id* | ***+*** | ***+*** | *-* | ***+*** | *-* |
| beta-lactam | NDM-1 | **+** | **+** | - | **+** | - |
| beta-lactam | SHV-12 | **+** | **+** | - | **+** | - |
| beta-lactam | TEM-1B | **+** | **+** | - | **+** | - |
| beta-lactam | LAP-2 | - | - | - | **+** | - |
| colistin | *mcr-9* | ***+*** | ***+*** | *-* | *-* | *-* |
| macrolide | *mph(A)* | *-* | *-* | *-* | ***+*** | *-* |
| phenicol | *catA2* | ***+*** | ***+*** | *-* | *-* | *-* |
| phenicol | *floR* | *-* | *-* | *-* | ***+*** | ***+*** |
| quinolone | *aac(6')-Ib-cr* | ***+*** | ***+*** | *-* | *-* | ***+*** |
| quinolone | *qnrS1* | *-* | *-* | *-* | ***+*** | *-* |
| quinolone | *qnrS2* | ***+*** | ***+*** | *-* | *-* | *-* |
| quinolone | *qnrB2* | *-* | ***+*** | *-* | *-* | *-* |
| quinolone | *qnrB6* | *-* | *-* | *-* | *-* | ***+*** |
| sulphonamide | *sul1* | ***+*** | ***+*** | *-* | ***+*** | *-* |
| sulphonamide | *sul2* | *-* | *-* | *-* | ***+*** | ***+*** |
| trimethoprim | *dfrA12* | - | ***+*** | *-* | ***+*** | *-* |
| trimethoprim | *dfrA27* | *-* | *-* | *-* | *-* | ***+*** |
| trimethoprim | *dfrA19* | ***+*** | ***+*** | *-* | *-* | *-* |
| rifampicin | *arr-3* | - | *-* | *-* | *-* | ***+*** |
| tetracycline | *tet(A)* | - | *-* | *-* | ***+*** | *-* |
| tetracycline | *tet(B)* | - | *-* | *-* | ***+*** | *-* |
