# Supplementary material for: Molecular investigation of an outbreak associated with total parenteral nutrition contaminated with NDM-producing Leclercia adecarboxylata
Source: BMC Infect Dis. 2021 Feb 28;21:235. doi: 10.1186/s12879-021-05923-0 (PMC7916303; doi:10.1186/s12879-021-05923-0)
Supplement: Supplementary file 3 — Additional file 3: S3 Table. Class 1 integron identified in the L. adecarboxylata 16,342 and 16,400 genomes. [file 12879_2021_5923_MOESM3_ESM.docx]

Supplementary Table 3. Class 1 integron identified in the *L. adecarboxylata* 16342 and 16400 genomes.

| **Genome** | **Class 1 integron array** | **In number** | **Reference** |
| --- | --- | --- | --- |
| 16342 | *IntI*1Δ-*aacA4* | In46 | HQ832472 |
| 16342 | *IntI*1Δ-*aadA2*-*qacE*Δ1-*sul1*-*orf5*Δ | In127 | EU089667 |
| 16400 | *IntI*1Δ-*aacA4* | In46 | HQ832472 |
| 16400 | *IntI*1Δ-*aadA2*-*qacE*Δ1-*sul1*-*orf5*Δ | In127 | EU089667 |
| 16400 | *IntI1-dfrA12-gcuF*Δ23 | In1982 | This study |
