# Supplementary material for: Molecular investigation of an outbreak associated with total parenteral nutrition contaminated with NDM-producing Leclercia adecarboxylata
Source: BMC Infect Dis. 2021 Feb 28;21:235. doi: 10.1186/s12879-021-05923-0 (PMC7916303; doi:10.1186/s12879-021-05923-0)
Supplement: Supplementary file 4 — Additional file 4: S4 Table. Incompatibility groups identified in the genomes of L. adecarboxylata included in the analysis. [file 12879_2021_5923_MOESM4_ESM.docx]

Supplementary table 4. Incompatibility groups identified in the genomes of *L. adecarboxylata* included in the analysis.

| Inc | 16342 | **16400** | **USDA-ARS-USMARC-60222** | **I1** | **R25** |
| --- | --- | --- | --- | --- | --- |
| **Q1** | *-* | *-* | *-* | ***+*** | *-* |
| **R** | *-* | *-* | *-* | ***+*** | ***+*** |
| **X3** | *-* | *-* | *-* | ***+*** | *-* |
| **FIA(HI1)** | *-* | *-* | *-* | *-* | ***+*** |
| **FII(Yp)** | ***+*** | ***+*** | *-* | *-* | *-* |
| ***HI2*** | ***+*** | ***+*** | *-* | *-* | *-* |
| ***HI2A*** | ***+*** | ***+*** | *-* | *-* | *-* |
